# The Brugada syndrome mutation A39V does not affect surface expression of neuronal rat Cav1.2 channels

**DOI:** 10.1186/1756-6606-5-9

**Published:** 2012-03-02

**Authors:** Brett A Simms, Gerald W Zamponi

**Affiliations:** 1 Department of Physiology and Pharmacology, Hotchkiss Brain Institute, University of Calgary, Calgary, Canada; 2 Department of Physiology and Pharmacology, University of Calgary, 3330 Hospital Dr., NW, Calgary T2N 4N1, Canada

**Keywords:** L-type calcium channel, Beta subunit, Brugada, Channelopathy, Traffic, Cav1.2

## Abstract

**Background:**

A loss of function of the L-type calcium channel, Cav1.2, results in a cardiac specific disease known as Brugada syndrome. Although many Brugada syndrome channelopathies reduce channel function, one point mutation in the N-terminus of Cav1.2 (A39V) has been shown to elicit disease a phenotype because of a loss of surface trafficking of the channel. This lack of cell membrane expression could not be rescued by the trafficking chaperone Cavβ.

**Findings:**

We report that despite the striking loss of trafficking described previously in the cardiac Cav1.2 channel, the A39V mutation while in the background of the brain isoform traffics and functions normally. We detected no differences in biophysical properties between wild type Cav1.2 and A39V-Cav1.2 in the presence of either a cardiac (Cavβ2b), or a neuronal beta subunit (Cavβ1b). In addition, the A39V-Cav1.2 mutant showed a normal Cavβ2b mediated increase in surface expression in tsA-201 cells.

**Conclusions:**

The Brugada syndrome mutation A39V when introduced into rat brain Cav1.2 does not trigger the loss-of-trafficking phenotype seen in a previous study on the human heart isoform of the channel.

## Background

Cav1.2 is an L-type voltage-gated calcium channel that is indispensible for proper function of organs including the brain and the heart [1]. Structurally, Cav1.2 channel complexes are composed of a pore-forming Cavα1 subunit, an accessory Cavα2δ subunit, and a Cavβ trafficking chaperone [2] which interacts with the Cavα1 subunit at the intracellular region linking the first two transmembrane domains [3-5]. Extensive alternate splicing of Cav1.2 between neuronal and cardiac backgrounds alters channel structure and function, as does the type of Cavβ subunit that is expressed in a given tissue [6-9]. Gain of function mutations in Cav1.2 channels may result in a multi-organ disease known as Timothy syndrome which is characterized by cardiac symptoms such as a prolonged Q-T interval, arrhythmias and sudden cardiac death (SCD); as well as immune dysfunction and autism [10]. A loss of Cav1.2 function on the other hand, can give rise to a heart specific disorder termed Brugada syndrome whose phenotype consists of a shortened Q-T interval, ventricular fibrillation and SCD [10]. Brugada syndrome has been associated with a gain of function in KCNE potassium channels [11], as well as a loss of function of Nav1.5 (15% of all cases) and Cav1.2/Cavβ (5% of all cases) [1,12]. How exactly increased Cav1.2 activity yields a disease phenotype in heart and brain, whereas reduced function selectively affects the heart is unknown, but may be explained by tissue-specific splice isoforms of the channel. Recent reports of splice isoform specific effects of mutations in P/Q-type and T-type calcium channels [13,14] may suggest that the tissue selective effect of Brugada syndrome mutations could be related to Cav1.2 channel sequences that are specific to the heart.

Recently a point mutation in the N-terminus of Cav1.2 (A39V) was identified in a patient with Brugada syndrome. This mutation resulted in a striking loss-of-function by way of disabled surface trafficking of the L-type channel complex [1]. The defective surface expression of A39V-Cav1.2 persisted upon coexpression of the cardiac Cavβ2b subunit, indicating that the effects of the mutation dominated over the well documented protective effect of Cavβ. This may be due to the possibility that intracellular linkers other than the I-II linker modulate surface expression of Cav1.2. Alternate splicing in the amino terminus of the channel can alter cell surface trafficking [6,15,16]. In addition an N-terminal splice variant specific to the heart termed the 'long variant', imparts PKC regulation upon the channel, while another shorter variant found in both heart and brain does not [17,18]. What is more important is that this second N-terminal variant is common to both the brain isoform used in our study and the cardiac channel used to test A39V-Cav1.2 previously. Other key sequence differences between cardiac and neuronal Cav1.2 variants do exist however, as do differences between human and rat channels, which are approximately 95% homologous (see Additional file 1: Figure S1).

The fact remains that the patient carrying A39V-Cav1.2 did not present with neurological symptoms raising the possibility that this mutation does not affect the sub-cellular trafficking of neuronal Cav1.2 channels.

To test this hypothesis we introduced the A39V mutation into rat brain Cav1.2 channels and examined its functional consequences in tsA-201 cells. Unlike in previous work with cardiac Cav1.2, we show that neuronal A39V-Cav1.2 retains Cavβ2b-dependent increases in surface expression, as well as total expression. We did not detect any biophysical differences between A39V-Cav1.2 and WT-Cav1.2 in the presence of either a cardiac or neuronal Cavβ subunit. We thus conclude that splice isoform differences between cardiac and neuronal Cav1.2 channels underlie the absence of a brain phenotype for the A39V Brugada mutation.

## Methods

### cDNAs/Mutagenesis

Wild type (WT) rat calcium channel subunit cDNAs encoding Cav1.2 (α1C), Cavβ1b and Cavα2δ1 subunits, as well as the pMT2 vector were generously donated by Dr. Terry Snutch (University of British Columbia, Vancouver, BC). Rat Cav1.2 has a polymorphism (glycine at amino acid position 57) adjacent to the A39V Brugada mutation locus which is not present in the human cardiac isoform. To facilitate comparison with previous work [1], we mutated rat Cav1.2 at position 57 to aspartic acid using QuickChange Site-Directed Mutagenesis Kit (Stratagene) as per manufacturer's instructions. The primer used for the G57D Cav1.2 mutagenesis was GGCAGGCAGCCATCGACGCCGCCCGGCAGGCC and its molecular complement. The A39V mutation was then constructed in both non-tagged and HA tagged Cav1.2 constructs using the primer AATGCAGCTGCAGGACTTGTCCCCGAGCACATCCCTACTCC. Following mutagenesis and cDNA preparation all clones were sequenced to verify the presence of desired mutations and overall sequence fidelity. The HA tagged version of Cav1.2 used has been previously described [19]. The Cavβ2b construct was generously donated by Dr. Henry Colecraft (Columbia University, New York, USA). GenBank™ accession numbers for the clones used are as follows: Cav1.2 [M67515], Cavβ1b [NM017346], Cavβ2b [AF423193.1], and Cavα2δ1 [AF286488].

### Tissue culture and transient transfection

Human embryonic kidney tsA-201 cells were grown and transiently transfected using the calcium phosphate method as described previously [3]. Transfection solutions for individual culture dishes contained a mixture of cDNA expression vectors, with the following quantities of cDNA expression constructs used: WT, or A39V calcium channel Cav1.2 subunit (3 μg), Cavβ subunit (3 μg), Cavα2δ1 (3 μg) and in addition for electrophysiology transfections, 0.25 μg pEGFP marker vector (Clontech). Non-tagged Cav1.2 clones were used for electrophysiology, while HA-tagged clones were transfected for all other experiments. Transfections which lacked a Cavβ subunit included 3 μg of pMT2 vector. Twelve hours post-transfection cells were washed once with PBS (pH 7.4), supplemented with fresh DMEM, and allowed to recover for 12 h. To prevent overgrowth for electrophysiology, cells were transferred to a 29°C incubator and maintained for 48-72 h prior to voltage-clamp recording. For immunoprecipitation/Western blot and immunofluorescence experiments cells were kept at 37°C for 48-72 h after PBS/DMEM treatment and grown to 75-85% confluence.

### Immunoprecipitation and Western blotting

Cultured tsA-201 cells were transiently transfected as described above with HA tagged channels for immunoprecipitation assays and were lysed with a modified RIPA buffer (in mM; 50 Tris, 130 NaCl, 0.2% triton X-100, 0.2% NP-40, 5 EGTA, pH 7.4). Lysis was carried out on ice for 15 min after which cells were centrifuged at 13, 000 rpm for 5 min at 4°C. Supernatants were then transferred to new tubes and solubilized proteins were incubated with 50 μl of Protein G beads (Pierce/Promega) and 1 μg of HA antibody (Roche) overnight while tumbling at 4°C. Total inputs were taken from whole cell samples representing 2.5% of the total protein and probed for alpha-actin (Sigma). Immunoprecipitates were washed once with the previously described modified RIPA buffer and a second time with a high salt RIPA buffer (in mM 500 NaCl, 50 Tris, 0.1% triton X-100, 0.1% NP-40, pH 7.4) and a final time with PBS (pH 7.4). Following washing, beads were aspirated to dryness and Laemmli buffer was added to samples before incubating at 96°C for 10 min. Eluted samples were loaded on the appropriate percentage Tris-glycine gel and resolved using SDS-PAGE. Samples were transferred to 0.45 μm PDVF membranes (Millipore) and western blot analysis performed using 1/1000 anti-HA (Covance), or 1/1000 anti-actin (Sigma). GE Healthcare horseradish peroxidase-linked secondary antibodies of appropriate species (mouse and rabbit) were used at 1/10000 dilution. Image J (National Institute of Health) was used to quantify the integrated density of protein on Western blots. For each blot the background signal was subtracted from experimental integrated densities to obtain sample values. Background subtracted values for HA signal were then divided by background subtracted actin signal to obtain the HA/actin ratio.

### Epifluorescence imaging

Cultured tsA-201 cells were transiently transfected as described above with HA tagged channels. Seventy two hours after transfection cells were fixed with 4% paraformaldehyde, and immunostained with anti-HA (1/1000, Roche). Alexa Fluor 594-conjugated goat α-rat IgG antibody (Molecular Probes, 1/1000) was used as the secondary antibody. Cells were imaged using a Zeiss LSM-510 Meta confocal microscope with a 40 × 1.2NA water immersion lens in the inverted position. The AF-594 antibody was visualized by excitation with a HeNe laser (543 nm) and emission detected using a 585-615-nm band pass filter. Image acquisition was performed with identical gain, contrast, laser excitation, pinhole aperture (fully open), scan size and laser scanning speed for all samples. Quantification of fluorescent signal was done following offline threshold adjustment with Image J. To obtain values for fluorescence/cell the total fluorescence per image was divided by the number of cells above threshold in that image.

### Cav1.2 voltage clamp recordings

Glass cover slips carrying cells expressing A39V or WT Cav1.2 channels (no HA tag) were transferred to a 3.5-cm culture dish (Corning) containing external recording solution consisting of 20 mM BaCl2, 1 mM MgCl2,10 mM HEPES, 10 mM Glucose and 136 mM CsCl (pH 7.4 adjusted with CsOH). Micro-electrode patch pipettes were pulled and polished using a DMZ- Universal Puller (Dagan Corporation) to a typical resistance of 3-5 MÙ. Internal pipette solution consisted of 110 mM CsCH3SO3, 20 mM TEA-Cl, 10 mM EGTA, 2 mM MgCl2 and 10 mM HEPES (pH 7.2 adjusted with CsOH).

Whole cell patch clamp recordings were performed in voltage-clamp mode using an Axopatch 200B amplifier (Axon Instruments) linked to a personal computer with pCLAMP software version 9.2. Series resistance was compensated by 85%, leak currents were negligible, and the data were filtered at 5 kHz. Individual pEGFP expressing cells were held at -100 mV. For steady state inactivation curves, we applied 4.5 s conditioning depolarizations, followed by a test pulse to +10 mV for 0.5 s. Individual sweeps were separated by 15 s. All stable cells with detectable inward current at 0 mV were used to calculate current density. Only those cells whose whole cell current voltage relationships could be fit with the modified Boltzmann equation, I = (1/(1 + exp^(-(Va-V)/S))^)*(V-E_rev_)*G_max_, where 'I' is current, 'V_a_' is half-activation potential, 'V' is membrane potential, 'E_rev_' is reversal potential, S is the slope factor, and 'G_max_' is slope conductance, were used for determination of voltage-dependent properties. As well, only cells whose steady-state inactivation could be fit by the Boltzmann equation, I/I_max _= A_2 _+ A_1_/(1 + exp^((V-Vh)/S)^), where 'I/I_max_' is normalized current, 'A_2_' is the non-inactivating fraction, 'A_1_' is inactivating fraction, 'V' is membrane potential, S is the slope factor, and 'V_h_' is half inactivation potential, were used to calculate voltage-dependent properties of steady-state inactivation.

### Data analysis

All electrophysiological data were analyzed using Clampfit version 9.2 (Axon Instruments) and fit in Origin 7 (Origin Lab Corporation). Image J was used to quantify the integrated density units (IDUs) of protein on Western blots as describe above. For quantification of fluorescent images Image J was used as described above yielding units of Arbitrary Light Units (ALUs). Statistical analyses for both biochemical and electrophysiological data were carried out using Origin 7. All sample means are reported +/- SEM. Statistically significant differences between means were assessed using student's *t*-test, or one-way ANOVA at 95% confidence level as appropriate.

## Findings & conclusions

Brugada syndrome mutations contribute to cardiac disease by shortening the Q-T segment of contraction, leading to arrhythmia and sudden cardiac death [10]. Functional changes that reduce Cav1.2 conductance can lead to Brugada syndrome, but so can mutations that reduce the number of channels in the cell membrane due to compromised cell surface trafficking. It was shown that a point mutation (A39V) in the N-terminus of a cardiac isoform of Cav1.2 severely limited membrane expression of the channel even upon coexpression of the ancillary Cavβ2b subunit [1]. This is unexpected considering that the Cavβ subunit promotes ER export and surface trafficking of the channel by binding the intracellular linker connecting domains I and II of the calcium channel α_1 _subunit [16,20]. Given that Brugada syndrome does not involve compromised brain function, we wondered if the A39V mutation might trigger a similar loss-of-trafficking phenotype in neuronal Cav1.2 channels.

**Figure 1:**
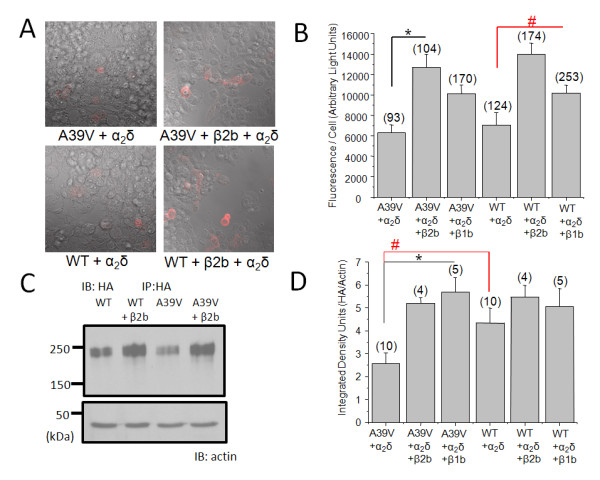
**Surface trafficking and total expression are the same for A39V-Cav1.2-HA and WT-Cav1.2-HA in the presence of β2b**. (**A**). β2b significantly increases the surface trafficking of A39V-Cav1.2-HA and WT-Cav1.2-HA in nonpermeablized tsA-201 cells. (**B**). Quantification of HA surface pool displayed as fluorescence per cell (arbitrary light units). β2b and β1b (Additional file 2: Figure S2A) significantly increase the fluorescence per cell of WT-Cav1.2-HA, while only β2b significantly increased the surface fluorescence of A39V-Cav1.2-HA (*p = < 0.05 and #*p *= < 0.05 by one-way ANOVA). Cavβ1b does not significantly increase the fluorescence per cell of A39V-Cav1.2 (*p *= > 0.05 by one-way ANOVA). (**C**). Total protein expression of A39V-Cav1.2-HA and WT-Cav1.2-HA from tsA-201 cell lysates expressed with and without β2b. (**D**). Quantification of A39V-Cav1.2-HA and WT-Cav1.2-HA total expression (integrated density units) with and without β2b/β1b (Additional file 2: Figure S2B). The data is expressed as a ratio of HA/α-actin. Both β2b and β1b significantly increase the expression of A39V-Cav1.2 (*p = < 0.05 by one-way ANOVA). A39V-Cav1.2 shows less expression than WT-Cav1.2 in the absence of Cavβ (#*p *= 0.04 student's *t*-test).

We first examined whether cell surface expression of A39V-Cav1.2 was different from that of wild type Cav1.2 (hereafter referred to as WT-Cav1.2) by staining for an external HA epitope on the channel in non-permeablized tsA-201 cells. We found that in the rat brain channel, surface expression of A39V-Cav1.2-HA (Figure 1A) was visible and that Cavβ2b significantly increased surface expression. Quantification of A39V-Cav1.2-HA fluorescence determined that the signal per cell (i.e., the surface pool of channels per cell), was significantly increased upon co-expression of a Cavβ subunit (Figure 1B). We found that WT-Cav1.2-HA and A39V-Cav1.2-HA were not differentially expressed at the cell surface (7064+/-1211 and 6298 +/-785 ALUs respectively). Coexpression of Cavβ2b significantly increased the surface pools of both WT-Cav1.2-HA (13087 +/- 964 ALUs, *p *= < 0.05 ANOVA) and A39V-Cav1.2-HA (12715 +/-1291 ALUs *p *= < 0.05 ANOVA). Cavβ1b was able to significantly increase the surface pool of WT-Cav1.2-HA (10145 +/-790 ALUs, *p *= < 0.05 ANOVA), and there was a strong trend towards increased surface expression of A39V-Cav1.2 (10109 +/-842 ALUs), however, this effect did not reach statistical significance (also see Additional file 2: Figure S2). Altogether, Figure 1A and 1B show that A39V-Cav1.2 is able to traffic to the cell membrane as well as WT-Cav1.2, and that the cardiac Cavβ2b subunit significantly increases surface expression of A39V-Cav1.2 in a neuronal background. Since previous work showed that Cavβ2b was unable to traffic the cardiac isoform of A39V-Cav1.2 to the cell membrane [1], the robust Cavβ-dependent trafficking observed in our experiments implies isoform specific effects for the A39V mutation. Splice variants of the Cav1.2 N-terminus have been shown to regulate cell surface expression of Cav1.2 in smooth muscle cells, indicating that this region of the channel may be involved in subcellular trafficking [6]. However, the N-terminus in our brain isoform has the same exon1b/2 composition as the channel construct used in previous work with cardiac A39V-Cav1.2 [1], and hence N-terminal variations cannot account for the observed differences between our findings and those reported previously. Several sequence differences between brain and cardiac isoforms do exist, however, one of which is a sequence insertion in the II-III intracellular linker of the brain isoform that has been hypothesized to augment protein binding in this region of the channel [21] (see Additional file 1: Figure S1). Moreover, the study by Antzelevitch and colleagues [1] utilized YFP tagged Cav1.2 channels containing exon 8a, whereas the Cav1.2 channels used in our experiments contained exon 8. It is possible that such splice isoform specific differences, or the attachment of a large YFP epitope could contribute to the observed differences in our findings compared to those reported previously. In our hands, A39V-Cav1.2 was able to traffic to the cell membrane as well as WT-Cav1.2, and the cardiac Cavβ2b subunit significantly increased surface expression of A39V-Cav1.2 in tsA-201 cells.

The Cavβ subunit has been shown to increase total expression of Cav1.2 by binding to the I-II intracellular linker of the channel and preventing ER associated degradation (ERAD) [22]. We therefore tested whether A39V-Cav1.2-HA total protein was increased upon coexpression of Cavβ2b and whether A39V-Cav1.2-HA expressed like WT-Cav1.2-HA without a Cavβ subunit (Figure 1C). Immunoprecipitation of the channels combined with semi-quantification against alpha-actin yield demonstrated that, in the absence of a Cavβ subunit, the integrated density of A39V-Cav1.2-HA (2.56 +/- 0.48 IDUs) was significantly less than WT-Cav1.2-HA (4.33 +/-0.66 IDUs, *p *= 0.04 by students *t*-test) (Figure 1D). Therefore, A39V-Cav1.2 is either produced to a lesser extent, or degraded more effectively than WT-Cav1.2. Since both Cav1.2 constructs were transfected identically and driven by the same constitutive promoter, the latter of these two possibilities appears more likely. Our data also reveal that coexpression of either Cavβ2b (5.20 +/-0.25 IDUs) (Figure 1C) or Cavβ1b (5.69 +/-0.62 IDUs) (Additional file 2: Figure S2B), results in a significant increase in A39V-Cav1.2-HA protein levels (*p *= < 0.05 by ANOVA) (Figure 1D). This confirms that the protective role of the Cavβ subunit is maintained in the A39V-Cav1.2 channel. Interestingly, total WT-Cav1.2-HA protein levels were increased upon coexpression of Cavβ1b (5.05 +/- 0.79 IDUs), but to a lesser degree than described by our lab previously for a Cav1.2. We attribute this to a different amino acid sequence in the N-terminus of the channel [22], perhaps suggesting that the N-terminus is involved in regulating Cav1.2 channel stability.

**Figure 2:**
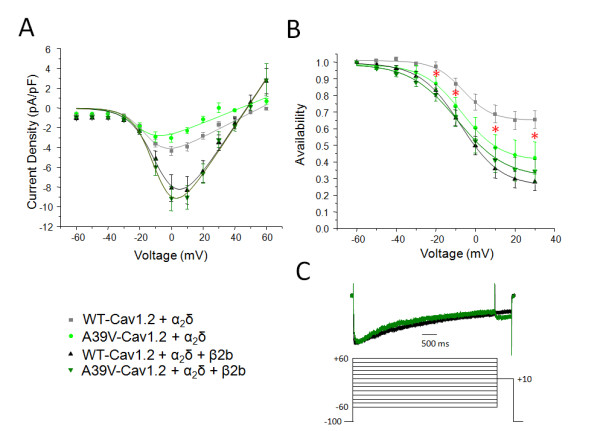
**The current voltage relation and steady-state inactivation of A39V-Cav1.2 is not significantly different from WT-Cav1.2 in the presence of β2b or β1b**. (**A**). The current-voltage relationships of WT and A39V-Cav1.2 with and without β2b. There is no significant difference in current density, or voltage-dependent properties when comparing WT-Cav1.2 (grey) and A39V-Cav1.2 (light green); or WT-Cav1.2 (black) and A39V-Cav1.2 (dark green) with Cavβ2b. Cavβ2b does significantly increase the current density of both WT and A39V-Cav1.2 (*p *= < 0.05 by one-way ANOVA). β1b also significantly increased current density of A39V-Cav1.2 (*p *= < 0.05 by one-way ANOVA) (Additional file 2: Figure S2). (**B**). Steady-state inactivation plots of WT and A39V-Cav1.2 with and without β2b. There is no significant difference in the steady-state of inactivation between WT-Cav1.2 and A39V-Cav1.2 in the presence of β2b or β1b (Additional file 2: Figure S2). The slope of steady state inactivation is increased for A39V-Cav1.2 when compared to WT-Cav1.2 in the absence of the Cavβ subunit (see Table 1) which significantly reduces the percentage of channels available at marked voltages (*) when compared to WT (*p *= < 0.05 by students *t*-test). (C) Voltage clamp protocol for inactivation curves, and sample traces of WT-Cav1.2 and A39V-Cav1.2 with Cavβ2b. Normalized traces of WT-Cav1.2 (black) and A39V-Cav1.2 (dark green) with Cavβ2b illustrating no significant difference in the time course of inactivation. Currents were evoked from a holding potential of -100 mV to various 4.5 s long conditioning potentials (ranging from -60 mV through +60 mV in 10 mV increments), followed by a test pulse to +10 mV for 0.5 sec.

We next evaluated whether the A39V mutation could alter the functional properties of the neuronal Cav1.2 isoform. In the absence of the Cavβ subunit WT-Cav1.2 exhibits a peak current density of -4.4 +/- 0.5, pA/pF at a test potential of 0 mV. As expected, co-expression of either Cavβ2b (-9.1 +/- 1.4 pA/pF, *p *= < 0.05 ANOVA), or Cavβ1b (-10.6 +/- 1.1 pA/pF, *p *= < 0.05 ANOVA) significantly increases the peak current density of WT-Cav1.2. The current-voltage relationship of A39V-Cav1.2 is not statistically different from WT-Cav1.2 in the presence of either Cavβ2b (Figure 2A), or Cavβ1b (see Additional file 2: Figure S2C). The A39V-Cav1.2 construct shows a peak current density of -3.3 +/- 0.5, pA/pF which does not differ from that of the WT channel and which is increased upon coexpression of Cavβ2b (-9.5 +/-1.6 pA/pF, *p *= < 0.05 ANOVA), or Cavβ1b (-8.3 +/- 1.0 pA/pF, *p *= < 0.05 ANOVA). Altogether, these data fit with our biochemical analysis in Figure 1. All other voltage-dependent properties of channel activation are similar in A39V-Cav1.2 and WT-Cav1.2 channels, with or without β subunit co-expression (Table 1). Moreover, the time course of inactivation (see Figure 2C) was not statistically different between A39V-Cav1.2 and WT-Cav1.2, with or without Cavβ subunit co-expression at all potentials tested between -10 mV and +30 mV (data not shown).

**Table 1:** Current densities and voltage-dependent properties of A39V-Cav1.2 without Cavβ, with Cavβ2b, or with Cavβ1b

	Current Density (pA/pF)	V_1/2 _Activation (mV)	Slope of Activation (mV)	V_1/2 _Steady State Inactivation (mV)	Slope of Steady State Inactivation (mV)
Cav1.2 + Cavα2δ	-4.4 +/- 0.5	-9.1 +/- 1.3	4.7 +/- 0.7	-5.4 +/-1.8	6.9 +/- 0.6#

A39V-Cav.2 + Cavα2δ	-3.3 +/- 0.5	-11.7 +/- 1.5	4.6 +/- 0.9	-7.3 +/- 1.9	11.8 +/- 1.4#

Cav1.2 + α2δ + Cavβ2b	-9.1 +/- 1.4 *	-3.8 +/- 0.8	10.2 +/- 1.4	-7.1 +/- 1.7	11.1 +/- 1.8

A39V-Cav1.2 + Cavα2δ + Cavβ2b	-9.5 +/- 1.3 **	-3.0 +/- 1.4	13.0 +/- 2.0	-5.2 +/- 2.8	11.5 +/- 1.2

Cav1.2 + Cavα2δ + Cavβ1b	-10.6 +/- 1.1 *	-9.0 +/- 0.8	8.8 +/- 1.2	-6.3 +/- 1.1	8.4 +/- 1.2

A39V-Cav1.2 + Cavα2δ + Cavβ1b	-8.3 +/- 1.0 **	-6.4 +/- 1.1	8.1 +/- 0.9	-6.8 +/- 1.6	9.3 +/- 0.8

- * indicates significant increase in current density when compared to Cav1.2 without a Cavβ subunit; *p *= < 0.05, ANOVA

- ** indicates significant increase in current density when compared to A39V-Cav1.2 without a Cavβ subunit; *p *= < 0.05, ANOVA

- Data expressed as mean +/- SEM. Current density was determined from a minimum of 24 cells per condition, from three separate transfections. All other values determined from aforementioned cells which fit the modified Boltzmann, or Boltzmann equations (> 11 cells/per condition)

- # indicates significant difference in slope of steady state inactivation; *p *= > 0.01, Students *T*-test

It has been demonstrated that mutations in the cardiac Cavβ2b subunit can affect inactivation of Cav1.2 in order to produce a Brugada phenotype [23]. Furthermore, the N-terminus of Cav1.2 has been shown to affect inactivation of the channel in a manner dependent on the Cavβ subunit [15]. We therefore tested whether the steady-state inactivation properties of A39V-Cav1.2 were different from those of WT-Cav1.2 in the presence of a Cavβ subunit. In the presence of either Cavβ2b (Figure 2B), or Cavβ1b (Additional file 2: Figure S2D) the steady-state inactivation properties of A39V-Cav1.2 are not significantly different from WT-Cav1.2. However, in the absence of the Cavβ subunit, A39V-Cav1.2 displays a significant increase in the slope of the inactivation curve (11.8 +/- 1.4 mV) which is significant when compared to WT-Cav1.2 (6.9 +/- 0.6 mV, p = < 0.01 students *t*-test). Furthermore, the A39V-Cav1.2 channel underwent a greater extent of total inactivation compared to the WT channel as denoted by red asterisks in Figure 2B (test potentials of -20,-10, +10, and +30 mV, p = < 0.05 by students *t*-test). This behavior of A39V-Cav1.2 could in principle be interpreted as a loss-of-function; however, as this occurs only in the absence of Cavβ, this effect will not likely manifest itself in native cells.

Here, we examined the Cavβ-dependence of the Brugada mutation A39V with regard to surface trafficking, total expression and function within the neuronal Cav1.2 isoform. Contrary to previous work on the cardiac isoform of Cav1.2 showing a loss of cell surface trafficking of A39V-Cav1.2 in the presence of Cavβ2b, we find that both cardiac Cavβ2b and neuronal Cavβ1b equally regulate the neuronal forms of mutant and WT Cav1.2 channels, and that the mutation does not alter the behavior of the neuronal channel in the absence of the Cavβ subunit. The concept of channel isoform-dependent effects of disease causing mutations is not without precedent [13,14] and in the case of the Brugada mutation A39V may perhaps explain why patients afflicted with this mutation do not exhibit a neuronal phenotype.

## Competing interests

The authors declare that they have no competing interests.

## Authors' contributions

BAS conducted and designed experiments, and contributed to the writing of the manuscript. GWZ directed the research and contributed to writing of the manuscript. All authors read and approved the final manuscript.

## Supplementary Material

Additional file 1 **Figure S1**. (A). Sequence alignment of the N-terminus of the two human A39V-Cav1.2 variants used in previous work [1] (accessions Z34815 & AJ224873) and the rat short N-terminus used in this study (M67515). Polymorphisms between rat and human channels are denoted by an 'X' below the amino acid position. Note however that the polymorphism (in red) present in the rat clone was mutated back to aspartic acid to match the human channels as addressed in the Methods. Both studies used short N-terminal splice variants of Cav1.2, which incorporate exon 1b. (B). Sequence alignment of the exon 8/8a segment of Cav1.2 used in each study. Note that YFP-containing exon 8a Cav1.2 was used for trafficking experiments in previous work [1], while exon 8 containing Cav1.2 was used in our study. Exon 8 (in red) is identical between human and rat. Polymorphisms detected between exon 8/8a are denoted by an 'X' below the amino acid position. (C). Sequence alignment of a portion of the II-III intracellular linker of the two human variants used in previous work [1] and the rat Cav1.2 channel used in our work. Note the inclusion of an inserted sequence in the rat channel which has been previously reported [21]. (D). Sequence alignment of the most divergent portion of the C-terminus of the two human cardiac sequences used in previous work [1] and the rat brain isoform used in our study. Click here for file

Additional file 2 **Figure S2**. (A). Cavβ1b increases the fluorescence per cell of WT-Cav1.2-HA (*p *= < 0.05 by one-way ANOVA), but not A39V-Cav1.2-HA (*p *= > 0.05 by one-way ANOVA). (B). β1b significantly increases the total protein expression of A39V-Cav1.2-HA (*p *= < 0.05 by ANOVA), but not WT-Cav1.2-HA. (C). Cavβ1b significantly increases current density of WT and A39V-Cav1.2 (*p *= < 0.05 one-way ANOVA). (D). Steady-state inactivation properties of A39V-Cav1.2 are the same as WT in the presence of β1b, for details see Table 1. Click here for file
