# Supplementary figures and images for: The Brugada syndrome mutation A39V does not affect surface expression of neuronal rat Cav1.2 channels
Source: Mol Brain. 2012 Mar 2;5:9. doi: 10.1186/1756-6606-5-9 (PMC3307476; doi:10.1186/1756-6606-5-9)

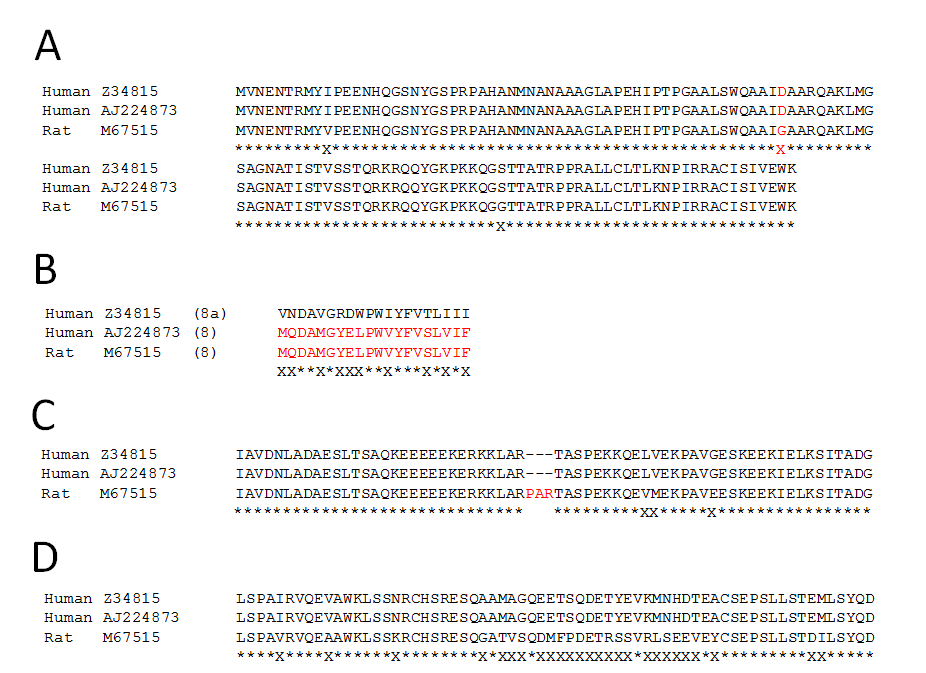

Supplement: Additional file 1 — Figure S1. (A). Sequence alignment of the N-terminus of the two human A39V-Cav1.2 variants used in previous work [1] (accessions Z34815 & AJ224873) and the rat short N-terminus used in this study (M67515). Polymorphisms between rat and human channels are denoted by an 'X' below the amino acid position. Note however that the polymorphism (in red) present in the rat clone was mutated back to aspartic acid to match the human channels as addressed in the Methods. Both studies used short N-terminal splice variants of Cav1.2, which incorporate exon 1b. (B). Sequence alignment of the exon 8/8a segment of Cav1.2 used in each study. Note that YFP-containing exon 8a Cav1.2 was used for trafficking experiments in previous work [1], while exon 8 containing Cav1.2 was used in our study. Exon 8 (in red) is identical between human and rat. Polymorphisms detected between exon 8/8a are denoted by an 'X' below the amino acid position. (C). Sequence alignment of a portion of the II-III intracellular linker of the two human variants used in previous work [1] and the rat Cav1.2 channel used in our work. Note the inclusion of an inserted sequence in the rat channel which has been previously reported [21]. (D). Sequence alignment of the most divergent portion of the C-terminus of the two human cardiac sequences used in previous work [1] and the rat brain isoform used in our study. [file 1756-6606-5-9-S1.TIFF]

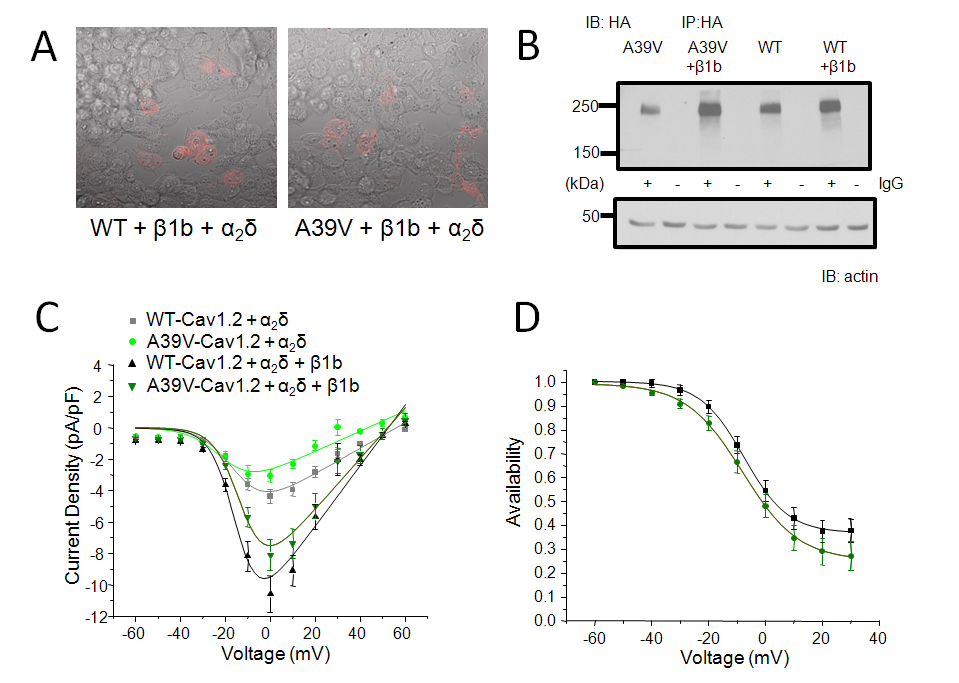

Supplement: Additional file 2 — Figure S2. (A). Cavβ1b increases the fluorescence per cell of WT-Cav1.2-HA (p = < 0.05 by one-way ANOVA), but not A39V-Cav1.2-HA (p = > 0.05 by one-way ANOVA). (B). β1b significantly increases the total protein expression of A39V-Cav1.2-HA (p = < 0.05 by ANOVA), but not WT-Cav1.2-HA. (C). Cavβ1b significantly increases current density of WT and A39V-Cav1.2 (p = < 0.05 one-way ANOVA). (D). Steady-state inactivation properties of A39V-Cav1.2 are the same as WT in the presence of β1b, for details see Table 1. [file 1756-6606-5-9-S2.TIFF]
